# Biologically induced spontaneous alginate gelation mediated by extracellular products of *Lactiplantibacillus plantarum*

**DOI:** 10.3389/fbioe.2026.1955981

**Published:** 2026-10-20

**Authors:** Weichen Gong, Solange Cisterna-Vergara, Miaya Kanazawa, Julio Villena, Keita Nishiyama, Haruki Kitazawa

**Affiliations:** 1 Laboratory of Animal Food Function, Graduate School of Agricultural Science, Tohoku University, Sendai, Japan; 2 Livestock Immunology Unit, International Education and Research Center for Food and Agricultural Immunology (CFAI), Graduate School of Agricultural Science, Tohoku University, Sendai, Japan; 3 Laboratory of Respiratory Immunology (LaRI), Division of Animal Immunology and Omics, International Education and Research Center for Food and Agricultural Immunology (CFAI), Graduate School of Agricultural Science, Tohoku University, Sendai, Japan

**Keywords:** alginate, bacteria-mediated functional biomaterials, lactic acid bacteria (LAB), lactobacilli, spontaneous gelation

## Abstract

**Introduction:**

Biological regulation of polysaccharide assembly represents an emerging concept in biofabrication. However, spontaneous gelation of natural polymers driven by bacterial extracellular metabolism remains largely unexplored. This study aimed to characterize a previously unrecognized alginate gelation phenomenon induced by *Lactiplantibacillus plantarum* without the intentional addition of conventional crosslinking agents.

**Methods:**

The effects of alginate concentration, bacterial cultivation time, and incubation temperature on gel formation were systematically investigated. The contributions of bacterial cells and cell-free supernatants were evaluated to identify the active fraction responsible for gelation. The role of acidification was examined using defined organic and inorganic acids, and the resulting gel microstructures, secondary Ca^2+^ crosslinking capability, processability, and bacterial viability during refrigerated storage were assessed.

**Results:**

Reproducible gelation was achieved using 1% (w/v) sodium alginate following 24 h of bacterial cultivation and subsequent incubation at 4 °C. Gel formation depended on bacterial growth, alginate concentration, and low-temperature treatment. Cell-free supernatants retained gelation activity, whereas bacterial cells alone failed to induce gel formation, indicating that extracellular bacterial products mediated the process. Although acidification alone was sufficient to initiate visible gelation, gels formed using defined acids exhibited microstructures distinct from those induced by bacterial extracellular products. Furthermore, the resulting gels remained processable, supported secondary Ca^2+^ crosslinking, and maintained high bacterial viability during refrigerated storage.

**Discussion:**

These findings demonstrate that extracellular products of *L. plantarum* can induce alginate gelation under mild conditions without intentionally added conventional crosslinkers. The distinct gel microstructures suggest that bacterial extracellular metabolism may influence gel architecture beyond acidification alone. This biologically induced gelation process provides a foundation for developing bacteria-directed biofabrication strategies, living biomaterials, and biologically engineered hydrogel systems.

## 1 Introduction

Hydrogels have attracted considerable attention in biomedical engineering, food science, drug delivery, and tissue engineering because of their high water content, biocompatibility, and tunable physicochemical properties (Kaur et al., 2024; Liu et al., 2025). Among various hydrogel-forming polymers, sodium alginate is one of the most extensively studied natural polysaccharides owing to its low toxicity, biodegradability, and mild gelation conditions (Frent et al., 2022). Conventionally, alginate gelation is achieved through ionic crosslinking with divalent cations such as Ca^2+^, although alternative approaches including covalent modification, photo-crosslinking, enzymatic reactions, and thermal treatments have also been developed to tailor gel properties (Gurikov and Smirnova, 2018). While these strategies have greatly expanded the applications of alginate-based materials, they generally require external crosslinking agents or chemical modifications. In contrast, biologically driven gelation strategies remain largely unexplored.

Among probiotic microorganisms, *Lactiplantibacillus plantarum* is one of the most widely used lactic acid bacteria in the food industry owing to its safety, robust environmental adaptability, and diverse health-promoting properties (Xie et al., 2025). *L. plantarum* was selected as the model organism in the present study because it is a robust acid-producing lactic acid bacterium (LAB) with broad relevance to probiotic and biomaterial applications. Importantly, the present study does not assume that the observed gelation phenomenon is unique to *L. plantarum*. Rather, this organism was used as a representative LAB model to establish and initially characterize the phenomenon (Xie et al., 2025). It has been extensively investigated for applications including probiotic delivery, intestinal health, antimicrobial activity, immune modulation, and functional food development (Wang et al., 2022; Tenea et al., 2026). To improve bacterial survival during processing, storage, and gastrointestinal transit, *L. plantarum* is frequently encapsulated within calcium alginate beads, one of the most widely used probiotic delivery systems because of their biocompatibility, mild preparation conditions, and protective effects on bacterial viability (Chen et al., 2025). In these encapsulation systems, alginate functions as a passive carrier, and gelation is typically achieved by external Ca^2+^ crosslinking prior to bacterial incorporation. Whether bacterial cultivation itself can actively induce alginate gelation, however, has received little attention.

During our previous study on the cultivation of bacteria within calcium alginate beads (Gong et al., 2026a), we evaluated the growth of *L. plantarum* under several culture conditions, including MRS medium containing 1% (w/v) sodium alginate. We unexpectedly observed that, after cultivation of *L. plantarum* at room temperature (25 °C), the culture system spontaneously transformed into an extrudable gel following subsequent incubation at 4 °C, without the addition of external crosslinking agents. This unexpected phenomenon suggested that bacterial cultivation altered the alginate system in a manner that promoted spontaneous gelation following low-temperature incubation. Such a phenomenon is of particular interest because it raises the possibility of establishing a bacteria-induced strategy for spontaneous polysaccharide gelation.

Recent studies have increasingly combined living bacteria with alginate-based hydrogels for applications including probiotic delivery, wound healing, and living biomaterials (Mao et al., 2026; Gong et al., 2026b; Sharma et al., 2026). In these systems, however, alginate gelation is typically achieved by conventional ionic crosslinking or other external gelation methods, while bacteria primarily function as encapsulated biological components rather than active regulators of gel formation. Previous studies have predominantly employed alginate as a passive encapsulation matrix, with gelation achieved using externally supplied calcium ions before or during bacterial incorporation. Whether bacterial cultivation itself can actively induce spontaneous gelation of externally supplied alginate remains largely unexplored (Frent et al., 2022). Therefore, the objective of the present study was not to optimize a hydrogel formulation but to establish and characterize this previously unrecognized biological gelation phenomenon.

In the present study, we systematically characterized this previously unreported gelation phenomenon. We investigated the conditions required for spontaneous gel formation, evaluated the processability and bacterial viability of the resulting gel, and performed an initial analysis of the contribution of bacterial acidification to gel formation. Our findings demonstrate that bacterial cultivation can induce spontaneous alginate gelation and suggest that extracellular bacterial products play an important role in determining the resulting gel architecture. This work provides a simple LAB-induced gelation strategy and establishes a foundation for future studies exploring how bacterial extracellular components regulate the formation and properties of polysaccharide-based biomaterials.

## 2 Methods

### 2.1 Bacterial strains and culture conditions

*Lactiplantibacillus plantarum* JCM1149 (Japan Collection of Microorganisms, RIKEN, Japan) was used as the primary model strain. Bacteria were cultured aerobically in de Man, Rogosa and Sharpe (MRS) medium (BD Biosciences, NJ, USA) at 37 °C under static conditions.

### 2.2 Preparation and sterilization of sodium alginate solutions

Sodium alginate (food grade; Fujifilm Wako, Osaka 541–0045, Japan) was dissolved in sterile deionized water to obtain solutions containing 0.5%, 1.0%, 1.5%, or 2.0% (w/v) alginate. To preserve the physicochemical properties of the polymer, heat sterilization was avoided because elevated temperatures may decrease the molecular weight and viscosity of sodium alginate. Instead, the freshly prepared solutions were sterilized by exposure to ultraviolet (UV) light in a biological safety cabinet for approximately 12 h under aseptic conditions.

Sterility of the UV-treated alginate solutions was confirmed using a culture-based assay. Briefly, calcium alginate beads prepared from UV-sterilized alginate without bacterial inoculation were incubated in sterile MRS broth under the same experimental conditions as those used for bacterial cultivation. No microbial growth was observed after 48 h of incubation at 37 °C, indicating that the UV sterilization method was sufficient for all experiments performed in this study.

### 2.3 Preparation of spontaneous alginate gels

Sodium alginate powder was directly dissolved in MRS broth to obtain the desired alginate concentration. The alginate-containing medium was sterilized by UV irradiation overnight before use as reported previously. For spontaneous gel preparation, 1.5 mL of alginate-containing MRS broth was transferred into each well of a 24-well plate. Subsequently, 5 μL of the bacterial suspension (approximately 1 × 10^7^ CFU/mL) was added to each well and thoroughly mixed to ensure homogeneous bacterial distribution throughout the medium. The cultures were incubated statically at 37 °C for the indicated time periods (6, 12, 24, or 48 h). Following bacterial cultivation, the culture plates were transferred directly to 4 °C and incubated for 1 h to induce spontaneous gelation. Unless otherwise specified, gels prepared using 1% (w/v) sodium alginate after 24 h of bacterial cultivation followed by 1 h of incubation at 4 °C were used in subsequent experiments.

### 2.4 Optimization of spontaneous alginate gelation

Gelation was evaluated using standardized calcium alginate beads as qualitative physical indicators. The indicator beads were prepared according to our previously established protocol (Gong et al., 2026a). Briefly, 2% (w/v) sodium alginate solution was extruded dropwise through a sterile 1-mL syringe into 1.5% (w/v) CaCl_2_ solution. The beads were allowed to crosslink for 30 min at room temperature and were subsequently washed three times with sterile PBS to remove excess calcium ions. This procedure generates approximately 20 beads per mL of alginate solution, corresponding to an estimated alginate volume of approximately 50 μL per bead.

For the gelation assay, one standardized calcium alginate bead was gently placed onto the surface of each sample. In preliminary validation, the beads sank in liquid MRS medium regardless of the presence or absence of bacterial cells. In contrast, after macroscopic gelation, the newly formed gel matrix prevented the bead from sinking. Samples in which the bead remained supported at or near the surface were therefore classified as gelled, whereas samples in which the bead sank into the medium were classified as non-gelled. Three technical replicates were included for each condition. This assay was used solely as a qualitative indicator of macroscopic gel formation and was not intended to quantify gel strength or mechanical properties.

### 2.5 Preparation of bacterial fractions and evaluation of spontaneous alginate gelation

To prepare bacterial fractions, *L. plantarum* was inoculated into 30 mL of MRS broth by adding 10 μL of an overnight bacterial culture and incubated statically at 37 °C for 18 h. The bacterial culture was then centrifuged at 6,500 rpm for 10 min at 4 °C. The resulting supernatant was collected and passed through a 0.22 μm syringe filter to obtain the cell-free supernatant (CFS). The bacterial pellet was washed twice with sterile phosphate-buffered saline (PBS) and resuspended in sterile distilled water to the original culture volume.

For evaluation of spontaneous alginate gelation, sodium alginate powder was directly dissolved into the bacterial suspension or the CFS to obtain a final concentration of 1% (w/v). Fresh MRS broth containing 1% (w/v) sodium alginate served as the negative control, whereas the whole bacterial culture before centrifugation was used as the positive control. All samples were subsequently incubated at 4 °C for 1 h, and gel formation was visually evaluated.

### 2.6 Acid-induced alginate gelation

To evaluate the contribution of acidification to spontaneous alginate gelation, alginate-containing MRS medium was acidified by titration with lactic acid, a 1:1 mixture of lactic acid and acetic acid, or hydrochloric acid (HCl). The acids were not added at predetermined molar concentrations. Instead, each acid was gradually added under continuous mixing while the pH was monitored until the target pH corresponding to that of the bacterial culture (approximately pH 4.3) was reached. Thus, final pH, rather than acid concentration, was used as the controlled experimental parameter. The exact final molar concentrations of the added acids were not determined. Sodium alginate powder was then directly dissolved into each solution to obtain a final concentration of 1% (w/v). After complete dissolution at room temperature, the samples were incubated at 4 °C for 1 h to induce gelation. The resulting gels were subsequently subjected to scanning electron microscopy (SEM) analysis and compared with gels prepared from the whole bacterial culture and the bacterial cell-free supernatant.

### 2.7 Scanning electron microscopy (SEM)

The microstructure of the bacteria-induced alginate gels was examined using scanning electron microscopy (SEM). Gel samples prepared from the whole bacterial culture before and after incubation at 4 °C, the bacterial cell-free supernatant (CFS), and acid-treated samples were collected and fixed in 2% (v/v) glutaraldehyde overnight at room temperature. The samples were subsequently dehydrated through a graded ethanol series (30%, 50%, 70%, 80%, 90%, 95%, and 100%, followed by a second immersion in 100% ethanol), with each dehydration step performed for 20 min.

After dehydration, ethanol was replaced with tert-butyl alcohol (t-BuOH), and the samples were freeze-dried. The dried specimens were sputter-coated with platinum and observed using a scanning electron microscope HITACHI SU8000 (Hitachi High-Technologies Corporation, Tokyo, Japan) under appropriate accelerating voltage.

### 2.8 Secondary calcium crosslinking

To evaluate secondary calcium crosslinking, bacteria-induced alginate gels were treated with 1.5% (w/v) CaCl_2_ solution for 20 min at room temperature. Gel diameter before and after calcium treatment was measured using manual measurement, and changes in gel size were compared between the bacteria-induced gel and the non-cultured alginate control.

### 2.9 Bacterial viability during storage

For bacterial viability analysis, bacteria-induced alginate gels were stored at 4 °C, 25 °C, or 37 °C for up to 7 days. At the indicated time points, 100 μL of gel was collected using a 1 mL syringe and transferred into 900 μL of sterile 1× PBS. The suspension was thoroughly dispersed by repeated pipetting and serially diluted 10-fold in sterile PBS. Aliquots (10 μL) of each dilution were spotted onto MRS agar plates and incubated overnight at 37 °C. Colony-forming units (CFU) were subsequently counted, and bacterial viability was expressed as CFU/mL.

### 2.10 Evaluation of gel processability

The processability of the bacteria-induced alginate gel was qualitatively evaluated by extrusion, secondary calcium crosslinking, direct writing, topical spreading, and washability. For extrusion analysis, the gel was loaded into a 1 mL syringe and extruded directly into distilled water to evaluate its continuity and shape retention. For secondary calcium crosslinking, the extruded gel was sprayed with 1.5% (w/v) CaCl_2_ solution and incubated for 20 min at room temperature to obtain a mechanically stabilized construct. For direct writing, the gel was manually extruded through a 1 mL syringe to generate predefined patterns prior to calcium treatment. The printed structures were subsequently fixed by spraying with 1.5% (w/v) CaCl_2_. For topical application, the gel was manually applied to the forearm skin of the first author to evaluate its spreadability. Washability was assessed by rinsing the gel under running tap water without mechanical scrubbing. Representative photographs were recorded throughout these procedures to demonstrate the processing characteristics of the gel.

### 2.11 Statistical analysis

All experiments were performed using three independent biological replicates (n = 3), and the results are expressed as mean ± standard deviation (SD). No technical replicates were included in the statistical analysis. Owing to the limited number of biological replicates, formal tests for normality were not conducted. Statistical significance was evaluated using either Student’s t*-test* for comparisons between two groups or one-way analysis of variance (ANOVA) followed by Tukey’s multiple comparisons test for comparisons among three or more groups, as specified in the corresponding figure legends. Significant differences were considered statistically significant at *p* < 0.05.

## 3 Results

### 3.1 L. plantarum induces spontaneous alginate gelation

During routine cultivation of *L. plantarum* in MRS medium containing 1% (w/v) sodium alginate, we unexpectedly observed a spontaneous transition of the alginate solution into a gel-like state following incubation at 4 °C. Freshly prepared alginate solution remained fluid after the same low-temperature storage, whereas the co-cultured sample became a homogeneous, extrudable gel that maintained its shape upon tube inversion (Figure 1A), indicating that bacterial cultivation was required for gel formation.

**FIGURE 1:**
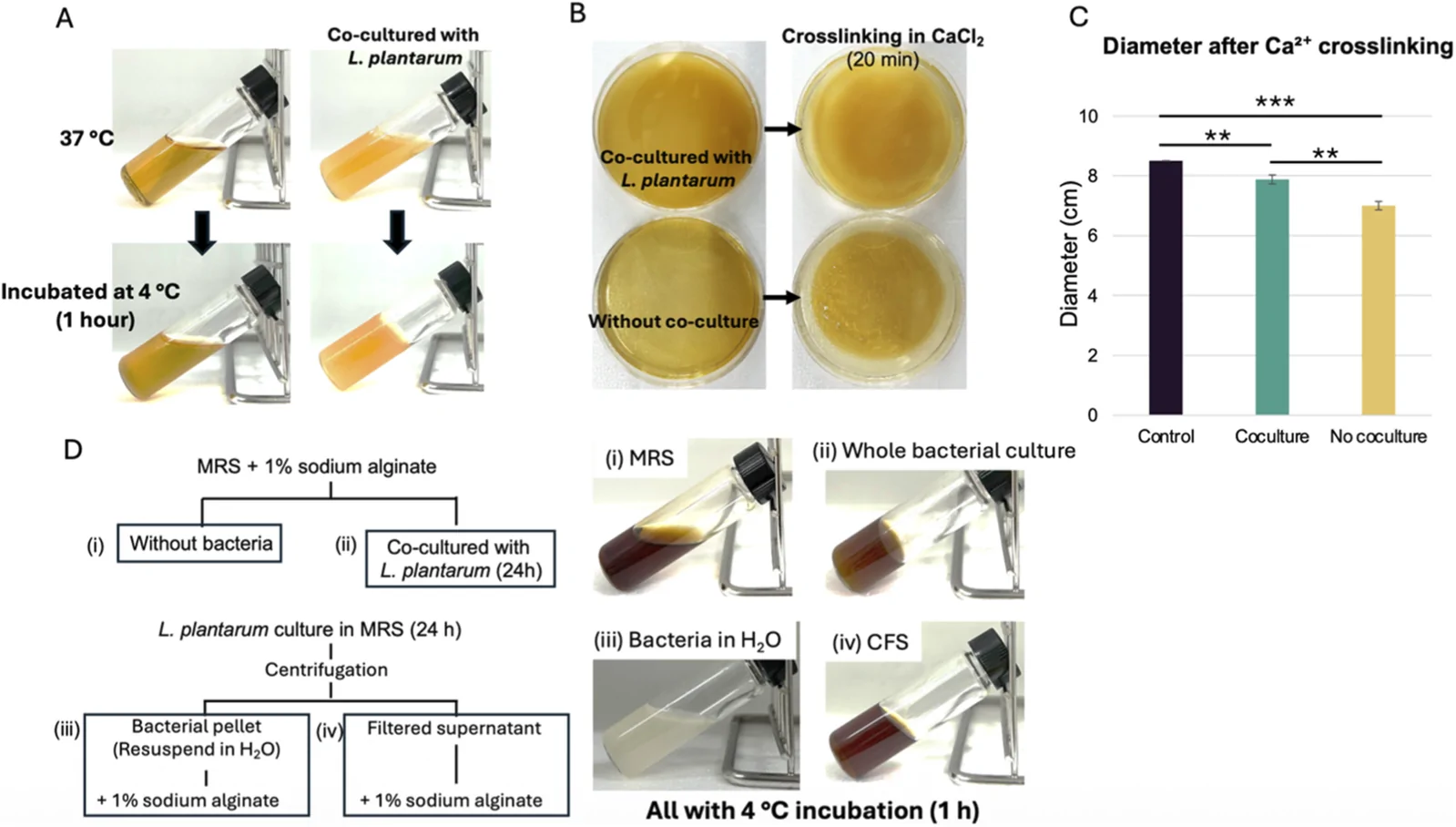
*Lactiplantibacillus plantarum* induces spontaneous alginate gelation. **(A)** Representative photographs showing the spontaneous transition of 1% (w/v) sodium alginate following co-culture with *Lactiplantibacillus plantarum*. Alginate without co-culture remained liquid at room temperature, whereas co-cultured alginate transformed into a hydrogel after storage at 4 °C for 1 h. Alginate without bacterial co-culture remained in the liquid state under the same conditions. **(B)** Macroscopic appearance of the hydrogel before and after CaCl_2_ treatment. The hydrogel generated by bacterial co-culture was readily converted into a mechanically stable gel by Ca^2+^ crosslinking, whereas alginate without co-culture exhibited greater shrinkage after the same treatment. **(C)** Quantitative comparison of gel shrinkage following CaCl_2_ crosslinking. Gel diameter was measured after 20 min of crosslinking. Data are presented as mean ± SD (n = 3). Statistical significance was determined using one-way ANOVA followed by Tukey’s multiple comparisons test. *p* < 0.01 (**), *p* < 0.001 (***). **(D)** Schematic illustration identifying the component responsible for hydrogel formation. Whole bacterial culture, bacterial cells resuspended in water, cell-free supernatant (CFS), and fresh MRS medium were separately mixed with 1% sodium alginate and incubated at 4 °C for 1 h. Hydrogel formation was observed only in samples containing bacterial metabolites.

To evaluate whether the resulting gel could undergo further ionic crosslinking, the co-cultured gel was treated with 1.5% (w/v) CaCl_2_ solution. The gel rapidly formed a mechanically stable construct while retaining its overall shape (Figure 1B). In contrast, alginate without bacterial co-culture exhibited more pronounced shrinkage after Ca^2+^ crosslinking. Quantitative analysis confirmed that bacterial co-culture significantly reduced gel contraction following crosslinking compared with the non-co-cultured control (Figure 1C), suggesting that spontaneous gelation altered the subsequent crosslinking behavior of alginate.

To identify the component responsible for gel formation, whole bacterial culture, bacterial cells resuspended in water, cell-free supernatant (CFS), and fresh MRS medium were separately mixed with sodium alginate. Gelation was reproduced by the whole culture and the CFS, whereas bacterial cells alone failed to induce gel formation (Figure 1D). These results indicate that LAB-derived extracellular components, rather than bacterial cells themselves, are associated with spontaneous alginate gelation under these conditions.

### 3.2 Gelation depends on alginate concentration and bacterial cultivation time

To determine the conditions required for spontaneous alginate gelation, sodium alginate solutions with different concentrations were co-cultured with *L. plantarum* for various cultivation periods prior to low-temperature incubation (Figure 2A). Gelation was evaluated using a calcium alginate bead flotation assay, in which successful gel formation prevented the bead from sinking into the sample. It should be noted that the calcium alginate beads used in the subsequent optimization assay were not involved in inducing spontaneous gelation, but served only as standardized physical indicators for distinguishing macroscopically gelled from non-gelled samples.

**FIGURE 2:**
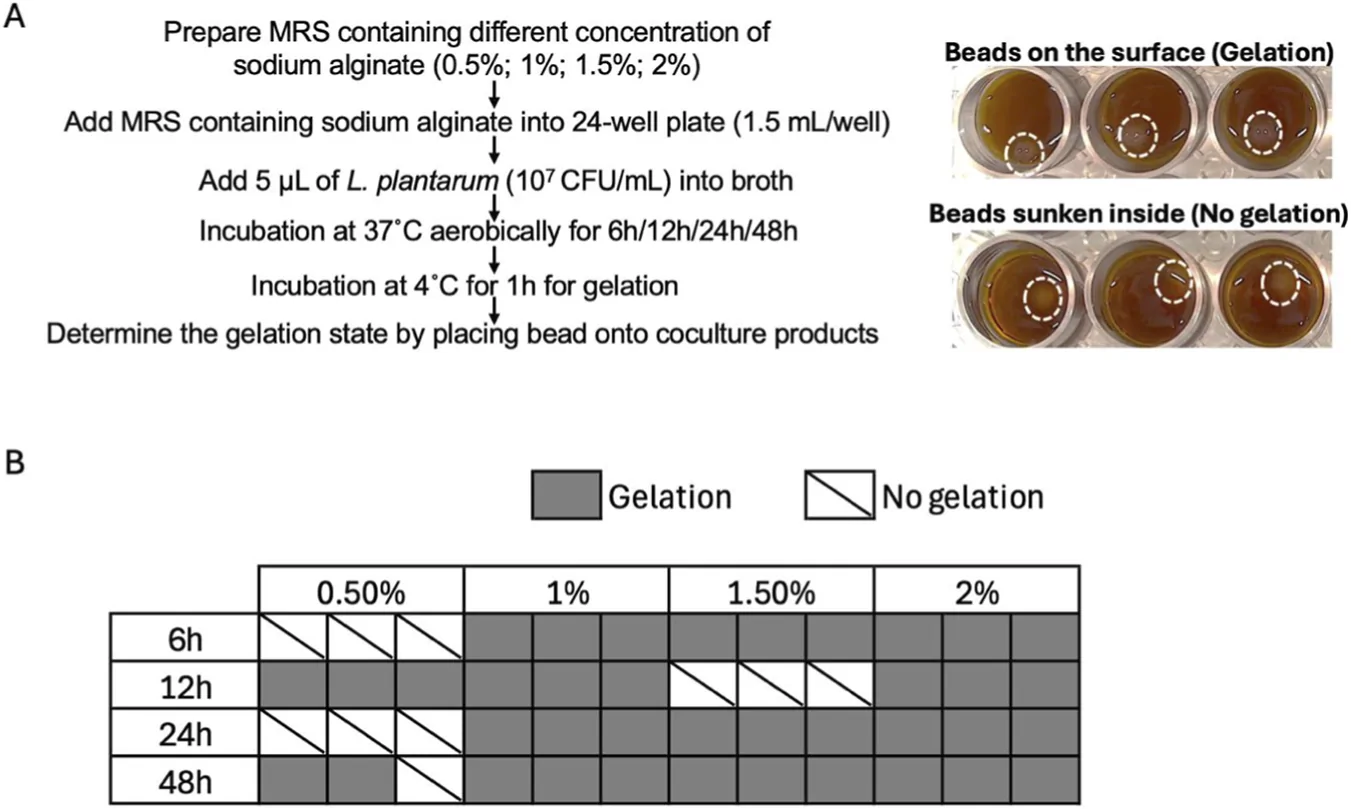
Gelation depends on alginate concentration and bacterial cultivation time. **(A)** Experimental workflow and principle of the qualitative bead-based gelation assay. Standardized calcium alginate beads prepared from 2% (w/v) sodium alginate were used solely as physical indicators. In non-gelled liquid samples, the bead sank into the medium, whereas after macroscopic gel formation the bead was supported by the gel matrix and remained at or near the surface. The bead itself was not involved in inducing spontaneous gelation. **(B)** Summary of hydrogel formation under different sodium alginate concentrations and bacterial cultivation times. Grey squares indicate successful hydrogel formation, whereas striped squares indicate the absence of gelation. Each condition was evaluated in triplicate, and the experiment was independently repeated twice with consistent results. Representative results from one independent experiment are shown.

Hydrogel formation was strongly influenced by both alginate concentration and bacterial cultivation time (Figure 2B). At an alginate concentration of 0.5%, gelation occurred only under limited conditions, whereas 1% alginate consistently produced stable gels after bacterial cultivation. Increasing the alginate concentration to 2% further broadened the conditions supporting gel formation. These findings indicate that spontaneous alginate gelation depends on both sufficient bacterial growth and an appropriate polymer concentration, providing practical conditions for reproducible gel preparation in subsequent experiments.

The dependence of gelation on alginate concentration further suggests that the formation of a continuous polymer network requires a sufficient density of alginate chains (Farrés and Norton, 2014; Jing et al., 2020). At low alginate concentrations, the distance between polymer chains is greater, reducing the probability of intermolecular interactions required for network formation. Consequently, although bacterial cultivation may modify the surrounding physicochemical environment, the available polymer content may be insufficient to establish a stable three-dimensional gel structure. Increasing the alginate concentration likely promotes chain entanglement and intermolecular association, thereby facilitating gel formation following bacterial cultivation and subsequent low-temperature incubation. These concentration-dependent differences are consistent with the existence of a threshold polymer concentration required for macroscopic network formation. Acidification may promote association of alginate chains, but a sufficiently high alginate chain density is still required to establish a continuous three-dimensional network. At 0.5% alginate, the polymer concentration may therefore be close to or below this threshold, resulting in gelation only under limited conditions. At intermediate concentrations, such as 1.5%, the system may remain sensitive to relatively small differences in the physicochemical environment generated during bacterial cultivation, including variation in final pH and extracellular composition. Increasing the alginate concentration further increases the probability of intermolecular association and broadens the conditions under which macroscopic gelation can occur.

### 3.3 The spontaneously gelled alginate exhibits versatile processing characteristics

Following spontaneous gelation, the material exhibited favorable processing characteristics for subsequent manipulation and shaping (Zhang and Wang, 2024). When extruded into water, the gel maintained a continuous filament immediately after extrusion before gradually dispersing over time, indicating its soft and deformable nature (Figure 3A). The gel could also undergo secondary ionic crosslinking by CaCl_2_ treatment (Zhang et al., 2024). After extrusion onto a substrate, spraying with CaCl_2_ solution rapidly converted the soft gel into a mechanically stable construct that retained its overall geometry (Figure 3B). Cross-sectional observation further confirmed that the gel maintained a well-defined three-dimensional structure after secondary crosslinking. The spontaneous gel could be directly written into customized patterns prior to CaCl_2_ treatment. As demonstrated by the formation of the letters “THU”, the printed structure remained intact after secondary crosslinking and could be manually adjusted before complete fixation (Figure 3C), demonstrating good moldability during processing.

**FIGURE 3:**
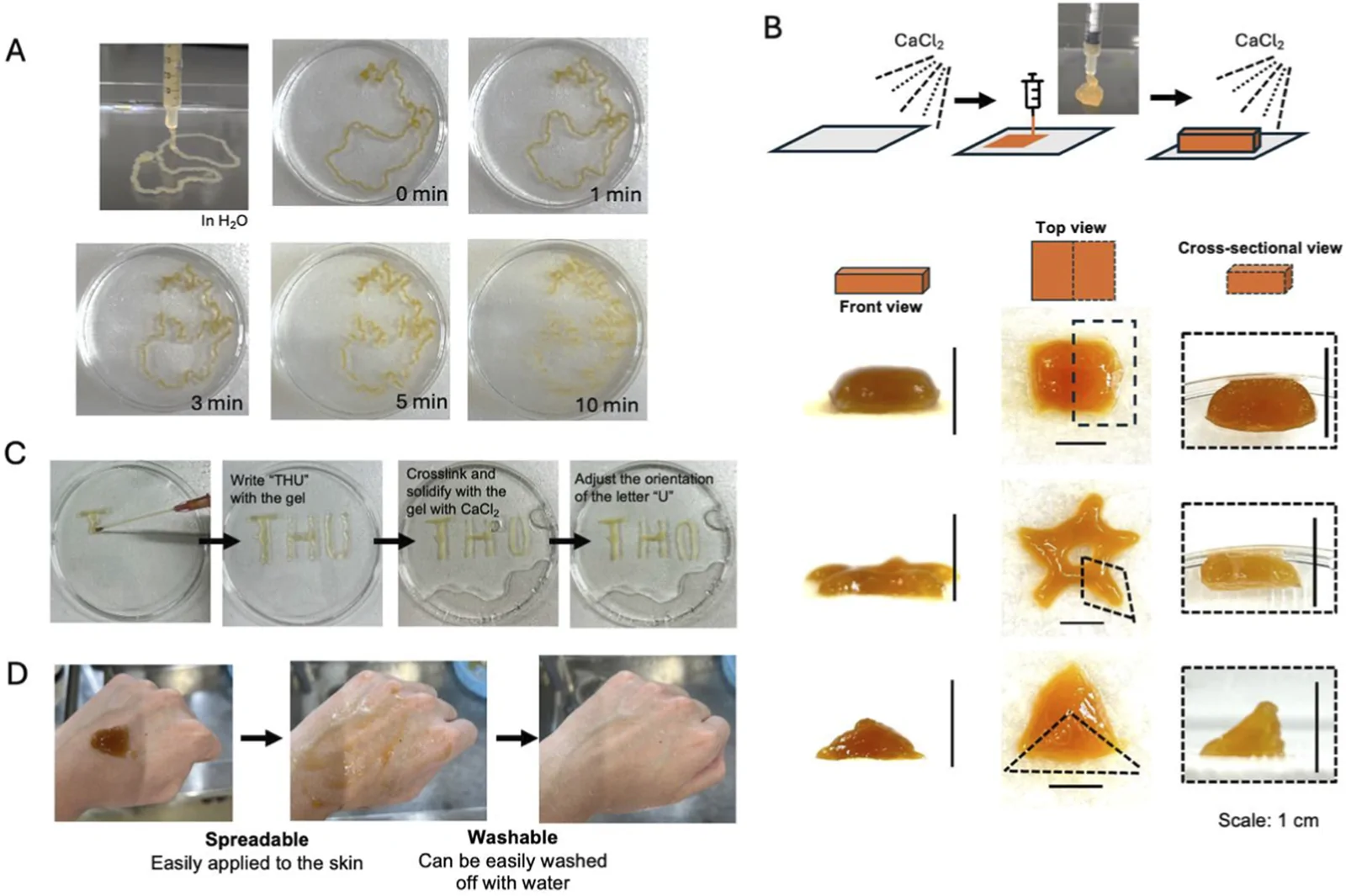
The spontaneously gelled alginate shows extrusion, writing, topical spreading, and secondary Ca^2+^ crosslinking. **(A)** Extrusion behavior of the spontaneously gelled alginate in water. The gel maintained a continuous filament immediately after extrusion but gradually dispersed over time, demonstrating its soft and extrudable characteristics. **(B)** Secondary crosslinking of the gel using CaCl_2_. The spontaneously formed gel was extruded onto a substrate and subsequently crosslinked by spraying CaCl_2_, producing mechanically stable structures. Representative front, top, and cross-sectional views demonstrate that the gel retained its three-dimensional shape after secondary crosslinking. **(C)** Demonstration of gel writing. **(D)** Evaluation of topical applicability. The gel was readily spread across the skin surface and could be completely removed by rinsing with water, demonstrating its potential for topical applications.

In addition, the gel was readily spread onto the skin surface to form a coating and could be completely removed by rinsing with water (Figure 3D), suggesting its potential suitability for topical applications. Collectively, these results demonstrate that the spontaneously gelled alginate can be readily processed by extrusion, secondary crosslinking, direct writing, and topical spreading without additional material modification.

### 3.4 Effect of storage temperature on bacterial viability after spontaneous alginate gelation

To evaluate the storage stability of the spontaneously gelled alginate for potential practical applications, bacterial viability was assessed after storage under different temperature conditions. Following gel formation, the alginate gels were stored at 4 °C, 25 °C, or 37 °C for up to 7 days, and bacterial survival was quantified by CFU enumeration after gel dissolution (Figure 4A). At 4 °C, *L. plantarum* remained highly viable throughout the entire storage period, with only a slight reduction in viable cell numbers after 7 days. Storage at 25 °C resulted in a gradual decrease in bacterial viability, whereas a more pronounced reduction was observed at 37 °C after 1 week of storage. To further evaluate changes in the gel microenvironment during storage, pH indicator strips were embedded within the gel matrix. Minimal color changes were observed throughout the storage period (Figure 4B), suggesting that the acidic microenvironment generated during bacterial cultivation was largely maintained under the tested storage conditions.

**FIGURE 4:**
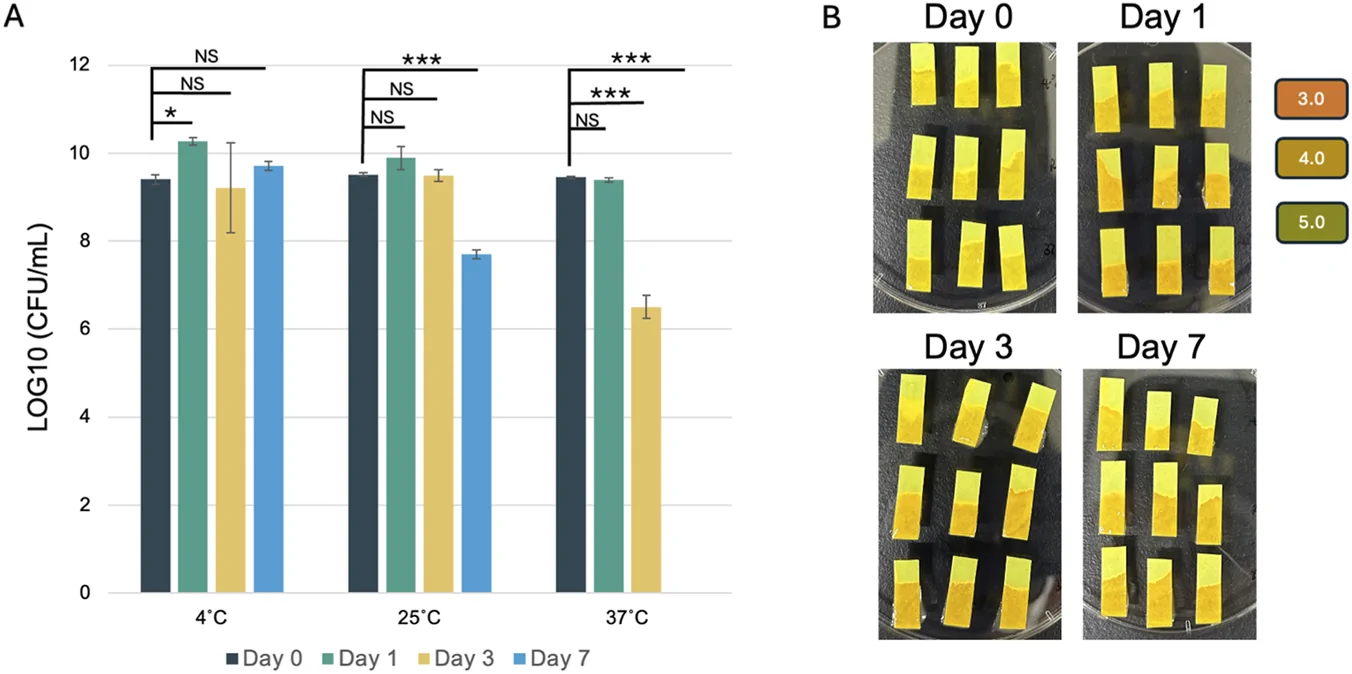
*Lactiplantibacillus plantarum* remains viable after spontaneous alginate gelation. **(A)** Viable bacterial counts in the hydrogel immediately after gelation (Day 0) and following storage at 4 °C, 25 °C, or 37 °C for 1, 3, and 7 days. Bacterial survival was quantified by colony-forming unit (CFU) enumeration after dissolving the hydrogel. Data are presented as mean ± SD (n = 3). Statistical significance was analyzed using one-way ANOVA followed by Tukey’s multiple comparisons test. *p* < 0.05 (*); *p <* 0.005 (***); NS, not significant. **(B)** Representative photographs showing the appearance of pH indicator strips embedded within the hydrogel during storage. These results qualitatively indicated that the acidic microenvironment remained relatively stable throughout storage.

Collectively, these findings suggest that storage at 4 °C provides the most favorable conditions for maintaining bacterial viability after spontaneous alginate gelation, supporting the potential storage and transportation of this bacteria-containing gel. From a practical perspective, maintaining bacterial viability during storage is essential for the future development of bacteria-containing biomaterials and probiotic-based products. The present results indicate that refrigeration at 4 °C is the most suitable storage condition among those examined, providing a practical basis for subsequent formulation development.

The different storage temperatures may have influenced bacterial physiology after gel formation (Cabello-Olmo et al., 2020). At 37 °C, L. plantarum is expected to remain metabolically active, and the progressive reduction in viable cell numbers after prolonged storage may reflect continued bacterial growth followed by nutrient depletion and accumulation of metabolic by-products within the gel matrix. In contrast, refrigeration at 4 °C is likely to substantially reduce metabolic activity, thereby slowing nutrient consumption and preserving bacterial viability during storage (Nedwell, 1999). Interestingly, the pH indicator remained largely unchanged throughout the storage period, suggesting that the acidic environment established during bacterial cultivation was relatively stable. This observation implies that factors other than further acidification, such as nutrient availability or metabolic stress, may have contributed to the decline in bacterial viability at elevated temperatures.

### 3.5 Acidification contributes to spontaneous alginate gelation but does not reproduce the characteristic hydrogel microstructure

To investigate the contribution of bacterial acidification to spontaneous alginate gelation, the microstructures of gels prepared under different conditions were compared by scanning electron microscopy (SEM). Alginate co-cultured with *L. plantarum* exhibited a dense structure before low-temperature incubation (Figure 5A). Following incubation at 4 °C, however, a porous three-dimensional network became apparent (Figure 5B). A comparable network structure was also observed in gels prepared using the cell-free supernatant (Figure 5C), supporting the involvement of extracellular components released during bacterial growth.

**FIGURE 5:**
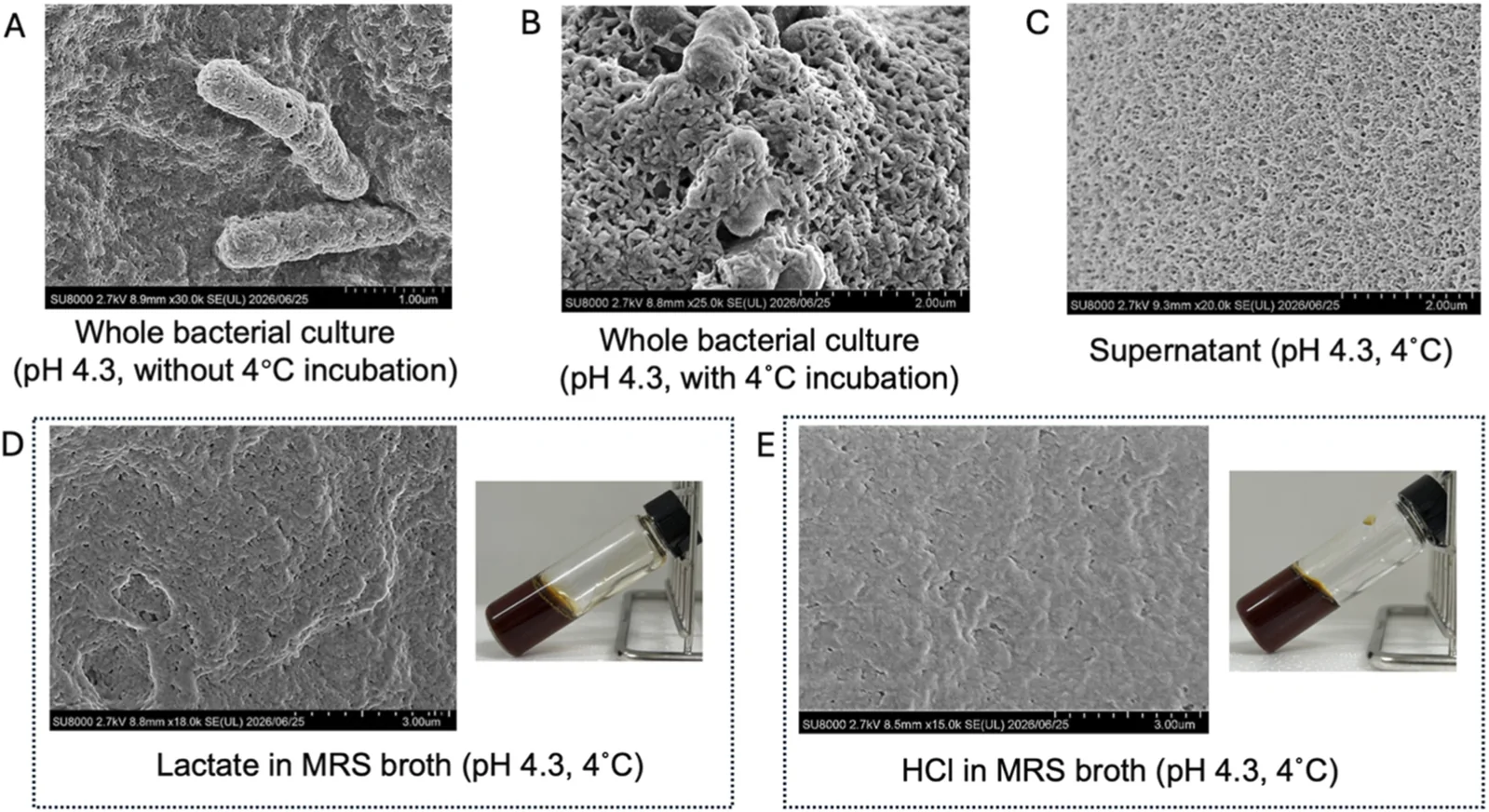
Acidification induces gelation but does not reproduce the microstructure formed by bacterial culture. **(A–C)** SEM images of alginate samples prepared from the whole culture at room temperature, the whole culture after incubation at 4 °C, and the cell-free supernatant after incubation at 4 °C. Low-temperature incubation following bacterial cultivation resulted in the formation of a porous three-dimensional network, which was also reproduced by the cell-free supernatant. **(D, E)** Alginate samples prepared by acidification with lactic acid or HCl (adjusted to pH 4.3). Although all acidified samples underwent visible gelation after incubation at 4 °C, SEM analysis revealed microstructures distinct from those generated by the bacterial culture and its supernatant, suggesting that acidification alone is insufficient to reproduce the characteristic hydrogel architecture.

Because *L. plantarum* primarily produces lactic acid during carbohydrate fermentation (Hu et al., 2019), sodium alginate was separately acidified using lactic acid or hydrochloric acid adjusted to the same pH (pH 4.3) as the bacterial culture. All acidified samples underwent visible gelation after incubation at 4 °C (Figures 5D,E). These results indicate that reaching a sufficiently acidic environment, rather than the presence of lactic acid specifically, is sufficient to initiate alginate gelation under the tested conditions.

SEM analysis, however, revealed that the chemically acidified gels exhibited relatively compact morphologies that differed markedly from the porous network generated by bacterial culture and CFS. These observations suggest that acidification represents a major physicochemical trigger for gel formation, whereas additional components present in the bacterial extracellular environment may modulate the extent of gelation and the resulting microstructure.

Mechanistically, lowering the pH is expected to increase protonation of alginate carboxylate groups (–COO^-^ to–COOH), thereby reducing electrostatic repulsion between polymer chains and promoting intermolecular association (Donati and Christensen, 2023). This process may favor the formation of alginic-acid-rich domains and ultimately facilitate development of a continuous gel network. The strong dependence of gelation on alginate concentration observed in Figure 2 is consistent with this model, because sufficient polymer-chain density would be required for protonation-driven intermolecular association to propagate into a macroscopic three-dimensional network. The subsequent requirement for incubation at 4 °C further suggests that acidification alone establishes a gelation-permissive state, whereas cooling promotes additional physical association or structural rearrangement of the protonated alginate chains. The precise molecular basis of this temperature dependence remains unresolved.

Nevertheless, the compact microstructures generated by chemically acidified samples differed markedly from the porous network observed in the bacterial-derived gels, indicating that acidification alone is insufficient to account for the characteristic gel architecture. The requirement for low-temperature incubation suggests that gelation is not solely governed by acidification but also involves temperature-dependent physical rearrangement of alginate chains. Although alginate gelation is known to be influenced by both pH and temperature, our SEM observations indicate that these physicochemical factors alone cannot explain the characteristic porous microstructure generated by bacterial culture, suggesting that additional extracellular bacterial products participate in this process.

## 4 Discussion

The present study should be regarded as an initial characterization of a previously unreported LAB-induced alginate gelation phenomenon rather than a comprehensive mechanistic investigation. Although our results demonstrate that bacterial cultivation promotes spontaneous alginate gelation and indicate that extracellular products are primarily responsible for this process, the specific components involved remain to be identified. Furthermore, while acidification was shown to trigger gel formation, the gels generated by lactic acid, acetic acid, or HCl exhibited microstructures distinct from those produced by bacterial culture or its cell-free supernatant, suggesting that acidification alone cannot fully explain the observed gel architecture. Future studies should therefore investigate the contributions of additional bacterial extracellular components, including EPS, proteins, extracellular DNA, and other metabolites, to clarify their individual and synergistic roles in gel formation.

### 4.1 Study limitations

A limitation of the present study is that only *L. plantarum* was examined. Different bacterial species produce distinct extracellular metabolites and possess diverse metabolic characteristics, raising the possibility that different microorganisms may generate gels with unique microstructures and physicochemical properties. Systematic comparisons among probiotic and non-probiotic bacteria may therefore establish a broader bacteria-induced gelation platform capable of producing polysaccharide gels with tunable structures and functions. Although the present study demonstrates that extracellular bacterial products are responsible for spontaneous gelation, the specific molecule(s) remain unknown. The cell-free supernatant contains numerous components, including organic acids, EPS, proteins, extracellular DNA, and other metabolites, making it difficult to identify the active factor without systematic biochemical fractionation. Identification of the gelation factor(s) therefore represents an important direction for future studies. Additionally, the requirement for low-temperature incubation suggests that spontaneous gelation involves temperature-dependent physical rearrangement of alginate chains in addition to acidification (Karakasyan et al., 2010). However, the molecular mechanism underlying this temperature dependence remains unknown and will require future investigation.

A further limitation concerns the physicochemical characterization of the spontaneous hydrogel. The primary objective of the present study was to establish a previously unrecognized bacteria-induced gelation phenomenon rather than to develop a fully optimized hydrogel material. Importantly, the gelation phenomenon itself was highly reproducible under standardized culture conditions. However, rigorous quantitative material characterization presents a different challenge because the hydrogel is generated through living bacterial metabolism in MRS medium. Although inoculum density, alginate concentration, cultivation time, and temperature were carefully standardized, slight biological variation among independent cultures inevitably resulted in small differences in the final pH (approximately ±0.2) and extracellular metabolite composition (Abka-Khajouei et al., 2022; Lee and Mooney, 2012). These biological variations do not affect the reproducible occurrence of gelation but may influence quantitative rheological or mechanical measurements.

### 4.2 Future perspectives and potential applications

Future studies will therefore establish chemically defined model systems using purified alginate, controlled acidification, and selected medium-derived components identified through systematic fractionation of MRS. Such reconstructed systems will allow the independent contributions of pH, alginate concentration, medium composition, and temperature to be examined under highly controlled conditions. They will also provide a more appropriate platform for systematic oscillatory rheology, mechanical testing, swelling analysis, degradation kinetics, water-retention measurements, and detailed mechanistic investigation.

Beyond its unique gelation mechanism, the spontaneously formed alginate gel may provide opportunities for several practical applications. First, because the gel is generated directly by bacterial cultivation while maintaining bacterial viability, it may serve as a promising platform for developing live probiotic bioinks for extrusion-based three-dimensional (3D) bioprinting (Wu et al., 2022). Unlike conventional bioinks that require bacterial encapsulation after hydrogel preparation (Sheet and Koley, 2019), the present strategy enables simultaneous bacterial cultivation and gel formation, potentially simplifying the fabrication process. Second, probiotic-derived metabolites, particularly the cell-free supernatant of *L. plantarum*, have been reported to promote wound healing and improve diabetic wound repair (Argañaraz et al., 2022). However, these products are generally administered in liquid form, limiting their residence time on wound surfaces and reducing sustained local activity. The spreadable alginate gel described in the present study may provide a simple carrier capable of prolonging local retention while preserving the bioactivity of bacterial metabolites, thereby offering potential advantages for topical wound management.

In addition, increasing attention has been devoted to topical probiotic formulations for oral and skin health, including probiotic gels for periodontal disease, oral mucosal disorders, acne, atopic dermatitis, and wound healing (Hillier et al., 2025; Łętocha et al., 2025; Deng et al., 2024). The spontaneous gelation strategy reported here may provide an alternative approach for preparing bacteria-containing topical formulations without conventional gel preparation procedures. More broadly, if different bacterial species or their extracellular products generate gels with distinct structures or material properties, bacteria-induced gelation may evolve into a versatile platform for engineering functional polysaccharide biomaterials with tunable biological and physicochemical characteristics.

Another attractive direction is the development of postbiotic-based hydrogels (Thorakkattu et al., 2022). Because the cell-free supernatant of *L. plantarum* retained the ability to induce alginate gelation in the present study, bioactive bacterial metabolites may be directly incorporated into the gel without requiring viable microorganisms. Such postbiotic hydrogels could provide a convenient platform for localized and sustained delivery of beneficial bacterial products while avoiding concerns associated with the stability, storage, or regulatory issues of live probiotics. This strategy may be particularly valuable for wound healing, tissue regeneration, and topical therapeutic applications.

More broadly, the present study raises the possibility of engineering hydrogel properties through bacterial extracellular products. Different bacterial species produce distinct combinations of organic acids, EPS, proteins, extracellular DNA, and other metabolites, which may differentially influence gelation behavior, microstructure, mechanical properties, and biological functions. Rather than relying solely on chemical crosslinkers, future studies may exploit these naturally produced extracellular components to tailor polysaccharide gels with desired structures and functionalities. Such a strategy could establish a versatile platform for designing bacteria-directed functional biomaterials.

## 5 Conclusion

In the present study, we describe a previously unreported phenomenon in which *L. plantarum* spontaneously induces alginate gelation following bacterial cultivation and subsequent low-temperature incubation. Unlike conventional alginate hydrogels, which generally rely on external ionic crosslinking using divalent cations such as Ca^2+^, the gel described here formed without the addition of any external crosslinking agent during the initial gelation process. The resulting material remained readily extrudable, could subsequently undergo Ca^2+^-mediated secondary crosslinking, and maintained bacterial viability during storage. These findings suggest that bacterial cultivation can serve as a simple biological strategy for generating functional alginate gels.

The mechanism underlying this phenomenon remains to be fully elucidated. Our results demonstrate that acidification initiates alginate gelation, while bacterial extracellular products shape or modulate the porous microstructure. Acidification alone was sufficient to trigger visible gelation; however, gels produced by lactic acid, acetic acid, or HCl exhibited microstructures that differed substantially from those generated by bacterial culture. These observations suggest that acidification contributes to gel initiation but cannot fully account for the formation of the characteristic porous network observed in the bacterial-derived gel. Additional extracellular components generated during bacterial growth are therefore likely to participate in determining the final gel architecture.

Besides its unique formation process, the spontaneously gelled alginate also exhibited several desirable material properties. The gel could be readily extruded, directly written into customized shapes, further stabilized by secondary Ca^2+^ crosslinking, and uniformly spread onto the skin surface before being easily removed with water. Such processing characteristics may facilitate future applications requiring simple fabrication procedures or localized delivery. Furthermore, the maintenance of bacterial viability after gelation indicates that the gel provides a suitable microenvironment for bacterial survival, potentially allowing continuous metabolic activity after gel formation.

Several limitations should also be acknowledged. The present study focused primarily on describing the spontaneous gelation phenomenon and its basic material characteristics, while the molecular mechanism responsible for gel formation was not fully investigated. Although *L. plantarum* was used as the model organism in the present study, whether the phenomenon is species-specific remains to be systematically determined. Given that acidification emerged as a major physicochemical driver of gelation, an important future question is whether other acid-producing microorganisms can similarly induce alginate gelation when a sufficiently low final pH is reached. Comparative studies across LAB species will be required to determine the generality of this phenomenon and whether species-dependent extracellular products influence the resulting gel architecture.

A further limitation concerns quantitative physicochemical characterization of the spontaneously formed gel. Dynamic oscillatory rheology, swelling behavior, degradation kinetics, and long-term mechanical stability were not systematically examined in the present study. Although the occurrence of macroscopic gelation was reproducible under standardized cultivation conditions, we observed modest variation in the extent of gelation among independent bacterial cultures. This biological variation was accompanied by small differences in final culture pH and is likely to reflect variation in extracellular metabolite composition. Because the present material is generated within a biologically active and chemically complex MRS culture system, such variation may directly influence quantitative rheological and stability measurements.

We therefore considered that rigorous determination of intrinsic material parameters would be more appropriately performed using a chemically defined reconstruction of the gelation system, in which polymer concentration, final pH, and individual medium-derived components can be independently controlled. Such a system would allow the contribution of each physicochemical parameter to be separated from biological batch-to-batch variation.

Future studies will therefore focus on identifying the extracellular components responsible for spontaneous alginate gelation. In particular, bacterial metabolites such as different organic acids, EPS, proteins, extracellular DNA, and other secreted molecules may contribute differently to gel formation and microstructure development. Comparative studies among different bacterial species may further reveal whether individual microorganisms produce characteristic gel architectures or confer distinct mechanical and biological properties. Such investigations may ultimately establish bacteria-induced alginate gelation as a versatile platform for engineering functional living biomaterials with tunable structures and properties. Although the present work primarily establishes a reproducible biological gelation phenomenon, important mechanistic questions remain unresolved, including the identity of the extracellular gelation factor(s), the molecular basis of the temperature dependence, and the physicochemical properties of the resulting hydrogel. These questions will be addressed in future studies using chemically defined model systems.

## Data Availability

The raw data supporting the conclusions of this article will be made available by the authors, without undue reservation.
